# Patient Journey for Triple-Negative Breast Cancer: Optimal Care Pathways vs. Reality of Care in Italian Breast Units

**DOI:** 10.3390/curroncol33020083

**Published:** 2026-01-31

**Authors:** Nicla La Verde, Luisa Brogonzoli, Maria Silvia Cona, Laura Cortesi, Elisabetta Iannelli, Eva Massari, Pietro Panizza, Roberto Papa, Maria Carmela Piccirillo, Elisa Sala, Valerio Mattia Scandali, Adele Sgarella, Laura Valentini, Rosaria Iardino

**Affiliations:** 1 Department of Oncology, Luigi Sacco Hospital, ASST Fatebenefratelli Sacco, 20157 Milan, Italy; 2 The Bridge Foundation, 20144 Milan, Italy; 3 Department of Medical Oncology, Azienda USL-IRCCS di Reggio Emilia, 42122 Reggio Emilia, Italy; 4 Department of Medical and Surgical Sciences, University of Modena and Reggio Emilia, 41125 Modena, Italy; 5 Italian Federation of Volunteer-Based Cancer Organisations (FAVO), 00187 Rome, Italy; 6 Italian Association for Cancer Patients, Relatives and Friends (AIMaC), 00187 Rome, Italy; 7 Breast Imaging Unit, IRCCS Ospedale San Raffaele, 20132 Milan, Italy; 8 Department of Staff, University Hospital of Marche Region, 60126 Ancona, Italy; 9 Clinical Trials Unit, Istituto Nazionale Tumori IRCCS Fondazione G. Pascale, 80131 Naples, Italy; 10 Hospital Medical Management, University Hospital of Marche Region, 60126 Ancona, Italy; 11 Department of Surgical Sciences, IRCCS Policlinico S. Matteo Foundation University of Pavia, 27100 Pavia, Italy; 12 Bridge for Future Research Centre, University of Pavia, 27100 Pavia, Italy

**Keywords:** triple-negative breast cancer, patient journey, optimal care pathways, multi-stakeholder method, co-created process, real-world evidence, Breast Units

## Abstract

Triple-negative breast cancer (TNBC) represents approximately 15% of all breast cancer diagnoses. Its heterogeneity and absence of targetable receptors make treatment particularly challenging, and it is burdened by a worse prognosis than other breast cancer subtypes. The absence of standardized care pathways may impede the accessibility of information for patients and caregivers. The present survey was conducted as part of a co-participatory process aimed at informing the development of a patient journey for TNBC. A group of statements was developed based on relevant literature, current guidelines, and good practice points (GPPs) for effective TNBC care and subsequently evaluated by a multi-stakeholder panel. Key steps in the care journey were then selected for the survey, and Coordinators of Italian BUs (65.4%) indicated the level of perceived importance and reported implementation of each item. Descriptive statistical analyses were employed. Despite some gaps concerning issues such as the involvement of general practitioners (GPs), data show agreement on perceived importance and generally adequate levels of reported implementation of core aspects of TNBC care, providing support for the development of an optimal care journey for triple-negative breast cancer patients grounded in real-world practice, which may help generate useful guidance where clear pathways are still lacking.

## Simple Summary

Treatment of triple-negative breast cancer patients poses significant challenges, primarily due to the lack of targeted therapies. The absence of clearly defined care pathways can result in unclear information for patients and differences in variability in care across facilities, a particularly important issue in treating this type of cancer. In this survey, Breast Units’ Coordinators assessed both the perceived importance and the reported implementation of key steps in triple-negative breast cancer management. Despite some gaps—mainly involving the role of general practitioners, waiting times, and the publication of Breast Unit lists by the various Italian regions—the results suggest general consensus and adequate reported implementation of core aspects of triple-negative breast cancer care. These findings support the development of an optimal care journey for triple-negative breast cancer patients grounded in real-world practice, which may aid in the creation of useful guidance where clear pathways are still lacking.

## 1. Introduction

According to the GLOBOCAN estimates, breast cancer (BC) was the most prevalent cancer among women worldwide in 2022 [1]. Within Italy, it accounts for approximately 30% of all female cancers, with an estimated 55,900 new cases recorded in 2023. Although a slight increase in incidence is anticipated in the coming years, mortality appears to be declining [2].

Triple-negative breast cancer (TNBC) constitutes approximately 15% of all BC diagnoses, corresponding to roughly 8000 new cases annually in Italy [3]. Compared with other subtypes of BC, TNBC has been shown to be associated with reduced overall survival (OS) and a high mortality rate. Furthermore, the disease is characterized by its high invasiveness, with propensity to distant metastases, and a relatively shorter relapse-free interval (RFI) [4].

TNBC is defined by the absence of oestrogen receptor (ER) and progesterone receptor (PR) expression, as well as the lack of human epidermal growth factor receptor 2 (HER2) overexpression [5,6]. These molecular features contribute to its poor prognosis. While these receptors have become the primary target of BC treatments, allowing advancements in the field, TNBC is not sensitive to endocrine or anti-HER2 therapies [7].

Current research is focused on the identification of novel prognostic markers, therapeutic targets, and combination strategies for both early-stage and advanced TNBC [8]. Poly (ADP-ribose) polymerase (PARP) inhibitors, such as olaparib and talazoparib, are among the recent advancements in targeted therapies for TNBC patients with germline pathogenic variants (gPV) in the *BRCA1/2* genes, and ongoing trials are evaluating their potential efficacy in non-*BRCA* pathways [9]. Furthermore, research is looking into additional therapeutic targets and combination therapies, including androgen receptor inhibitors, cyclin-dependent kinase (CDK) inhibitors, inhibitors of the PI3K/AKT/mTOR signalling pathway, and tyrosine kinase inhibitors (TKIs) [10,11,12,13,14,15,16]. Moreover, immunotherapy and immune checkpoint inhibitors, particularly programmed cell death protein 1 (PD-1) and programmed death-ligand 1 (PD-L1) inhibitors, have demonstrated variable outcome improvements in selected patient populations [17,18,19,20,21].

Notwithstanding these advancements, current treatment options continue to largely rely on chemotherapy plus or minus immunotherapy [22]. 

The limitations in the available treatment options, coupled with the heterogeneity of TNBC, result in a lack of specific targeted therapy for the disease. Moreover, regional and centre-based variations may affect aspects related to TNBC care. For example, Mirza et al.’s retrospective cohort study on cancer treatment centres in England emphasized regional differences in time to chemotherapy for patients treated with surgery and chemotherapy for TNBC [23]. Focusing on Italian Breast Units (BUs), Tinterri et al. conducted a survey that revealed significant variations in *BRCA* testing practices, which hold predictive and prognostic information for TNBC [24]. The authors reported that Breast Units affiliated with Senonetwork accounted for more than 95% of women who are treated every year in Italy for BC.

Such variability, paired with the absence of clear and comprehensive information related to the care journey of TNBC patients, may negatively affect patient experience and care outcomes.

The present survey was conducted with the aim of informing the development of a structured TNBC patient journey (PJ), which could take into account the ideal pathways of care, as well as the challenges, barriers, and difficulties in their implementation. In light of the challenges posed by the limited therapeutic options available for TNBC patients, the overall process focused on identifying key elements to support the development of specific care journeys tailored to TNBC. In the absence of targeted therapies, the presence or absence of a germline BRCA pathogenic variant (gPV) was selected as the primary factor on which specific patient journeys were differentiated.

In particular, the objective of the survey was to ascertain the level of agreement, in terms of both perceived importance and reported implementation, with respect to the elements deemed to be good practice in the care and management of patients with TBNC. By leveraging these data, it was possible to create a more precise and factual patient journey, grounded in the reality of Italian Breast Units, so that each patient could potentially refer to this document in their care journey and have access to the same range and availability of services across all Breast Units.

The PJ is a structured representation of a person’s care experience within the healthcare system, encompassing their journey from the initial presentation of health needs to follow-up, including clinical, organizational, and experiential dimensions, offering a comprehensive and holistic perspective. This approach allows for an integrated analysis of access to services, continuity of care, and critical points in the care journey. Specifically, the PJ for TNBC comprises the stages of screening adherence, diagnosis, definition of the multidisciplinary treatment plan, treatment (surgery, systemic therapy, and/or radiotherapy), follow-up, and long-term surveillance. The analysis of this journey facilitates the provision of support to patients and caregivers while also identifying diagnostic delays, inequalities in access, and organizational critical issues, thereby supporting the improvement of the effectiveness and equity of care.

The document represents an effort to address the complexity and uncertainty associated with TNBC. The PJ described should not be considered as a document comparable to, nor as a replacement for, clinical guidelines. Rather, it is a proposal intended to provide suggestions and support for the definition of specific care pathways for TNBC and to illustrate the care journey of TNBC patients. Furthermore, the development of a patient journey for a specific condition, such as TNBC, also entails the recognition of potential limitations, including the limited number of specialized centres and the possibility that they may not be located in close geographical proximity to patients.

Within this framework, the study aims to examine the reality of TNBC care in Italian BUs: by comparing real-world experiences with evidence-based recommendations and clinical guidelines, the survey aims to contribute to the identification of optimal care journeys for TNBC.

## 2. Materials and Methods

The approach and analysis employed in the development of the Statements and in the survey followed a previously established methodological framework, adapted to the present clinical context.

### 2.1. Survey Construction and Survey Aims

The initial phase entailed the establishment of a scientific group, encompassing breast oncologists, breast radiologists, breast surgeons, geneticists, representatives of patients’ associations, health policy experts, and economic-health researchers, with the objective of creating the survey’s statements.

The group’s work focused on reviewing the relevant literature and existing guidelines to identify key elements of TNBC care. To further develop the multidisciplinary approach, an additional group of key stakeholders was created. This panel included all professionals collaborating within the BU, namely psycho-oncologist, nutritionist, gynaecologist, fertility expert, pathologist, nurse, nuclear medicine specialist, plastic surgeon, radiotherapist–medical oncologist, general practitioner (GP), bone specialist, cardio-oncologist, and palliative care specialist. These figures were interviewed to identify good practice points (GPPs), with the aim of complementing the limited existing guidelines on TNBC care.

The literary review, interviews, and resulting GPPs allowed us to identify a set of 117 statements, which were then organized into five areas of interest, namely prevention, diagnosis, patient intake, therapy, and follow-up, with an additional section addressing the composition and meeting frequency of the multidisciplinary team.

The voting process for the statements, aimed at the scientific validation of the proposed optimal care journey, was based on the principles of the Delphi methodology, recognized for its effectiveness in achieving informed consensus among experts in complex areas, as reported in Diamond et al. [25]. This modified approach involved three steps:

- Members of the scientific group and key stakeholders evaluated the relevance and applicability of each of the 117 previously defined statements, using a pre-determined rating scale to express their level of agreement.
- A consensus threshold was established, typically defined as the agreement of at least 75% of the participants on the relevance of a statement, in line with standard clinical content validation procedures.
- To streamline the process and facilitate consensus, the sessions were supported by expert methodologists. In instances where a consensus could not be reached, the statements were discussed, revised based on expert feedback, and subjected to a re-voting cycle. In the event that consensus was not reached after three rounds, the statement was classified as belonging to an area of uncertainty or non-relevance for the working group, ensuring that the final set was robust, clinically applicable, and widely shared.

Subsequently, the approved statements were summarized into 24 core aspects of TNBC management based on the following criteria: first, avoiding redundancy among Statements by condensing overlapping items; second, reducing the overall number of items to enhance the feasibility of the survey; and third, ensuring alignment with the aim of the survey, with a focus on verifying organizational and management aspects rather than clinical/therapeutic issues, which were considered to be already addressed in dedicated guidelines.

The statements resulting from the application of these criteria were included in a survey sent to 156 BUs affiliated with Senonetwork, an Italian non-profit organization that comprises healthcare professionals, promoting compliance with national and European guidelines in Italian Breast Centres. Accordingly, Breast Units are required to meet specific criteria for inclusion within the network, contingent on the number of new cases per year (at least 150 cases per year) and quality requirements. From a regulatory and organizational perspective, Breast Units are considered comparable entities: regardless of whether a unit serves a large metropolitan area or a smaller local population, it is expected to provide the same core set of practices and to adhere to the same national guidelines. Consequently, further stratification or comparison across units would not be expected to yield additional interpretable or meaningful differences beyond those already captured by the analysis of reported implementation levels.

The survey aimed to assess the level of agreement among clinicians and the degree of implementation of the statements in Italian BUs, thereby providing further clinical and evidence-based support for the optimal TNBC care journey previously identified. It did not include the participation of patients, nor questions pertaining to personal patient data; rather, the statements derived from best practice points, clinical guidelines (national and international), national regulations, and diagnostic and therapeutic care pathway (PDTA) frameworks.

The survey included questions on the composition and functioning of the multidisciplinary team, as well as on prevention, diagnosis, patient intake, therapy, and follow-up pathways.

Firstly, with regard to the multidisciplinary team, respondents were asked to indicate which professions are represented within the team and how frequently meetings are held (e.g., weekly or bi-weekly).

Concerning prevention, the survey investigated the role of GPs in the early detection of patients at higher risk based on family history and the use of preventive strategies in identifying potential TNBC cases at an early stage.

With regard to diagnosis, the modalities through which TNBC is initially identified and staged were analysed: these include the use of mammography, ultrasound, and other imaging techniques, as well as the employment of PET scans, *BRCA* testing, and the issue of waiting times.

Patient intake procedures represented another key theme: questions pertained to the presence of the Case Manager, intake pathways across various specialists, access to fertility preservation (e.g., social egg freezing), waiting times for services, nutritional assessment, coordination between the equipe and GPs, and the list of active BUs. The involvement of patient and caregiver associations was also assessed.

The section on therapy focused on the main treatment options offered to patients, including surgical approaches, the availability of clinical trials, and the use of hormonal therapies.

Finally, for follow-up, the survey examined how post-treatment management is organized: this included the scheduling and structure of follow-up visits, the adoption of intensive follow-up protocols when indicated, and the availability of dedicated follow-up services.

The survey comprised a 24-statement questionnaire, with additional sub-statements for a total of 46, built on the six main areas of interest, divided as follows:

- 2 statements on the multidisciplinary team;
- 2 statements on prevention;
- 4 statements on diagnosis (11 including sub-statements);
- 8 statements on patient intake (15 including sub-statements);
- 4 statements on therapy;
- 4 statements on follow-up (12 including sub-statements).

These statements reflected practices that should represent a baseline standard applicable to all Breast Units, in order for the patient journey to be relevant nationwide, regardless of local organizational or regional differences.

In order to respond to the questions posed in the survey, participants were required to express their level of agreement or disagreement using a 5-point Likert scale. Each statement was analysed according to two subscales. The first subscale measured the perceived importance of the statement, ranging from (1) “of no importance” to (5) “extremely important”. The second subscale measured the degree of reported implementation of the statement in clinical practice, ranging from (1) ‘not implemented at all’ to (5) ‘extremely implemented’.

The survey was administered with the computer-assisted web interviewing (CAWI) method, utilizing a web-based program specifically designed for the management of research, surveys, and customer satisfaction studies. It was available online from 4 December 2024 to 4 February 2025. The survey was sent to the Coordinators of all the BUs recognized by Senonetwork, who served as the primary reference figures for the survey and, owing to their coordinating role, were able to consult with other relevant healthcare professionals when necessary to address questions requiring specific expertise. No patients, caregivers, or other stakeholders were asked to participate in the survey.

### 2.2. Data Analysis

The questionnaire was disseminated to a total of 156 centres certified by Senonetwork. The responses received were screened to eliminate any duplicates, preferring those provided by the centre’s representative where available, otherwise selecting the first response given. The final sample comprises 65.4% (N = 102) of the BUs contacted.

The present study exclusively employed descriptive statistical analyses, encompassing both measures of central tendency (mean scores, mode, and median) and measures of dispersion (standard deviation and interquartile range (IQR)), whilst refraining from the formulation of a priori assumptions. In order to account for potential structural and regional influences, the sample was stratified by Region and further analyses were conducted, which showed that regional context had no meaningful impact on the reported implementation of practices: observed differences were minor and not statistically significant.

All analyses were conducted using IBM SPSS Statistics (Version 28), employing built-in procedures.

The subsequent analysis of the scores was guided by the criteria outlined in the following section:

- Importance of the statement subscale: It was determined that ratings of 4, i.e., “important” or higher, indicated a strong consensus, while ratings of 3, i.e., “quite important” or lower, indicated a weak consensus. The level of consensus regarding the perceived importance of each statement was quantified by calculating a mean score for the five macro-areas of interest, as well as an overall score.
- Degree of implementation subscale: scores of 4, i.e., “properly implemented” or above, were considered to indicate a satisfactory level of reported implementation; a score of 3, i.e., “enough implemented”, indicated a moderate level; and a score of 2, i.e., “slightly implemented” or below, reflected a poor level of reported implementation. As with the importance subscale, a mean score was calculated for each macro-area and for the total.
- In order to identify items that may require additional efforts or targeted programs, the difference between perceived importance and reported implementation was examined. For the purpose of emphasizing the findings, a discrepancy greater than 0.75 was arbitrarily defined as indicating a substantial critical issue in the implementation of the statements. In contrast, discrepancies between 0.25 and 0.75 were indicated as less severe concerns.

The following interpretations of the results focused primarily on the mean score values.

## 3. Results

Overall, the results indicate a broad consensus regarding the perceived importance of the statements included in the survey, with no individual statement considered unimportant or of minor relevance. Conversely, reported levels of implementation were found to be comparatively lower and exhibited greater variability than perceived importance. However, slight to poor implementation was observed only for two statements concerning the role of general practitioners in the systematic review of medical history for BRCA gPV familiarity and their coordination with Breast Units. Consequently, these statements also exhibited a significant gap between perceived importance and reported implementation. A similar discrepancy was observed in relation to issues related to waiting times and the publication of Breast Unit lists by the respective regions.

### 3.1. Demographics

Respondents comprised 102 BUs’ Coordinators, corresponding to 65.4% of the total of the invites sent to the BUs recognized by Senonetwork. All Italian regions, with the exception of Calabria and Molise (which do not have any BUs in Senonetwork’s list), were represented; for half of the regions, the representativity of the sample is over 75% (see Table 1). However, some regions present a low number of BUs (e.g., Valle d’Aosta with one BU, Marche with two BUs), and therefore, despite their full representation, they exert no statistically significant influence on the results.

More than half of the respondents (60%, number of respondents = 61) were surgeons, whereas oncologists represented 30% of the sample. With respect to the number of patients, 61% (number of respondents = 62) of BUs reported treating between 100 and 500 patients in the last year, while 36% (number of respondents = 37) of them were responsible for the care of over 500 patients.

Two statements pertained specifically to the multidisciplinary team (see Table 2). Both aimed at verifying standards shared at the national and European level in Italian Breast Units, namely concerning the presence of key professionals within the Breast Unit and the frequency of BU meetings.

With respect to the composition of the BU team, the results emphasized the presence of key professionals defined by national and EUSOMA European standards in the core team in almost all cases: the breast surgeon and oncologist participated in 100% of cases, while the radiologist and pathologist exceeded 90% (number of respondents = 96 and 95). Other specializations were not as frequently incorporated into the core team: the cardiologist, physiatrist, nutritionist, palliative care physician, bone expert and dentist show a presence ranging from 3.9% to 21.6%; however, in several cases, their participation in the multidisciplinary team was facilitated where necessary through the presence of these figures in the facility or through structured collaboration with other centres.

In the majority of cases (87.3%, number of respondents = 89), in accordance with national and EUSOMA Breast Centre requirements, the multidisciplinary team convenes on a weekly basis, while the remaining 12.7% (number of respondents = 13) meet every two weeks.

### 3.2. Importance and Implementation Levels

In general, the trends in the mean values relating to the perceived importance and reported implementation of the statements appear to be almost identical, with some values perfectly aligned. This finding indicates a high degree of consistency between what is considered relevant for the care of patients and the actual provision of services. All the results of perceived importance and reported implementation for each Statement are shown in Table S1 (Supplementary File).

When the standard statistical measures (mean scores, medians, modes, standard deviations and IQRs) for perceived importance and reported implementation for each of the five areas (see Table 3) are observed, a high perception of importance emerges for all phases of the care journey: the mean scores range between 4.32 (for patient intake) and 4.51 (for diagnosis), and the mode and median are consistently equal to 5, indicating an almost unanimous consensus on their value. The interquartile ranges (IQRs) for perceived importance are relatively narrow (1 point across all areas), further highlighting strong agreement among respondents.

However, the degree of reported implementation appears to be lower and more variable with respect to that of perceived importance, with averages ranging between 3.45 (prevention) and 4.41 (diagnosis); furthermore, a higher standard deviation is observed for all areas, indicating significant differences in the practices adopted. The IQR for implementation is also wider for the areas of prevention and patient intake (2 points), suggesting more diverse experiences and operational challenges within these areas.

Specifically, diagnosis emerges as the area perceived as overall most important and best implemented, while prevention and patient intake demonstrate the most significant disparities between perceived importance and reported implementation, indicating operational criticalities.

These discrepancies are also apparent in therapy and follow-up, albeit to a lesser extent.

The data indicate an overall perception of importance across all aspects, and no particular criticalities with respect to reported implementation, with the exception of two statements. Moreover, the data also underscore the necessity for targeted strategies in the initial stages of the care journey.

#### 3.2.1. Importance

In order to provide further clinical support to the selected aspects of TNBC management, the survey assessed the level of perceived importance attributed by the BUs’ Coordinators to each statement.

No individual statement was regarded as either unimportant or of minor importance, while a total of six statements were assigned an average score below 4; however, it is worth noting that all statements were deemed to be of some importance (mean > 3). These statements are reported in Table 4.

With regard to the diagnosis phase, the statement ‘PET may be recommended for patients with triple-negative carcinoma at clinical stage ≥ II’ is not considered to be of particular importance.

In the patient intake phase, statements falling below the threshold of 4 pertain to the presence of specific specialist clinical interventions from the outset, namely consultation with a cardiologist, the presence of a bone specialist, and the dental assessment in cases where anti-blastic treatment is scheduled. This may be attributable to the intervention of designated professionals during particular phases of the treatment trajectory, rather than at the inception of the process.

For therapy, hormone therapy in the field of gynaecological health is not considered especially important.

In terms of follow-up, the statement that does not score above 4 points of perceived importance is related to the follow-up management of the patient being entrusted to the oncologist, with possible management by the GP one year after diagnosis for low-risk breast cancer.

#### 3.2.2. Implementation

The proposed optimal care journeys for TNBC were then compared with the real-life context of care to verify their reported implementation within the different Italian BUs. The results indicate an overall high level of reported implementation, with no statement considered completely unimplemented (score below 2.00). A total of two statements were identified as having an average score below 3. The first statement (1) obtained an average score of 2.70, and the second (12) obtained an average score of 2.97. These statements are placed between the categories of “poorly” and “slightly” implemented.

Both statements relate to the role of the GP with respect to their role in the systematic medical history review for familiarity with BRCA gPV and the coordination with the BU.

#### 3.2.3. The Gap in the Statements: Perceived Importance vs. Reported Implementation

In order to analyse the degree to which the reported implementation in real-life settings of care mirrored the importance attributed to key aspects of TNBC management, the gap between the two dimensions was examined.

With regard to the prevention phase, the first statement, concerning the implementation of a systematic medical history review by the GP for familiarity with BRCA gPVs, presents a gap > 1, indicating criticalities in the reported implementation compared to the importance conferred.

With respect to the diagnosis phase, there are no substantial gaps, and the mean values of perceived importance and reported implementation demonstrate a high degree of similarity.

Only one statement exhibits a slight gap, with a value greater than 0.25. This statement pertains to the subject of waiting times for tests (a maximum of two weeks for cases characterized by rapid disease progression).

Two statements in this phase present a negative gap, i.e., reported implementation greater than perceived importance (see Section 3.2.4).

In the initial patient intake phase, a number of statements are observed that require further consideration due to the presence of a gap greater than 0.75, indicating a substantial discrepancy between the importance conferred to the statements and their reported implementation.

These statements address frequently critical aspects, such as the reduction in waiting times (also observed in the diagnosis phase), coordination between the BU and the GPs, and the periodic updating and publication of the recognized BUs list by the regions. In the therapy phase, the statement referring to hormonal therapy presents a slight gap (greater than 0.25) in terms of its reported implementation, which follows an estimate proportionally similar to the importance given (which is rated as low, below 4). A similar observation can be made in relation to the statement concerning clinical trials, which also presents a minor gap > 0.25; however, both gaps are minimal and thus do not signify critical issues.

Finally, for follow-up, the possibility of management by a GP one year after diagnosis (for low-risk breast cancer), which is not considered to be of particular importance, also exhibits a discrepancy of >0.25.

The statements pertaining to follow-up services (fertility counselling, genetic counselling, bone health counselling, nutritional education counselling, cardiological counselling, education on symptoms of cancer recurrence), which are all considered of great importance, also exhibit a gap > 0.25.

**Figure 1:**
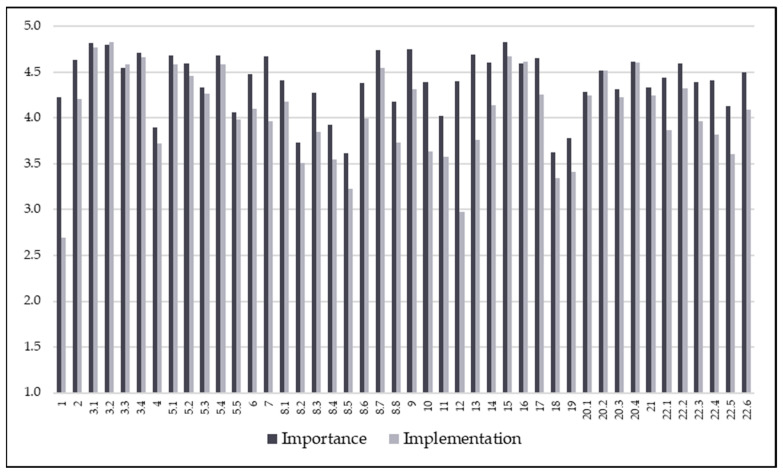
The chart illustrates the difference between the perceived importance of each statement and its reported implementation. The *x*-axis provides a list of the questionnaire items, while the *y*-axis shows the points of the 5-level Likert scale. Dark grey bars are used to indicate the perceived importance of each statement (ranging from (1) “not important at all” to (5) “extremely important”), whereas light grey bars are used to show the level of reported implementation (ranging from (1) “not implemented at all” to (5) “fully implemented”).

The perceived importance and reported implementation of the statements are illustrated in Figure 1.

In general, statements exhibiting a gap greater than 0.75 highlight a critical issue, emphasizing areas where the reported implementation significantly falls short in the importance attributed to them. These statements are reported in Table 5.

The areas of concern involve the role of GPs in prevention and case management, the issue of waiting times, and the publication of BUs lists by respective regions. In particular, in the context of prevention, there is an apparent lack of implementation of the systematic review by GPs to assess family history of BRCA gPVs, despite respondents recognizing the importance of this practice: while there is broad consensus on the pivotal role of GPs as “first referrers”, there seems to be a deficit in preventive measures undertaken by these practitioners, thereby limiting the timely involvement of at-risk relatives in screening and prevention programs.

Furthermore, in the context of patient intake, the coordination between the multidisciplinary team and the GP scored a mean < 2 in reported implementation, and a gap > 1 between perceived importance and reported implementation, signifying a discrepancy between the perceived importance and the reported implementation of the practice.

The other two statements exhibiting a relatively large discrepancy (≥0.75) pertain to waiting times and to the updating of the BU lists by regions; these will be analysed in greater detail in the subsequent Section 4.

**Figure 2:**
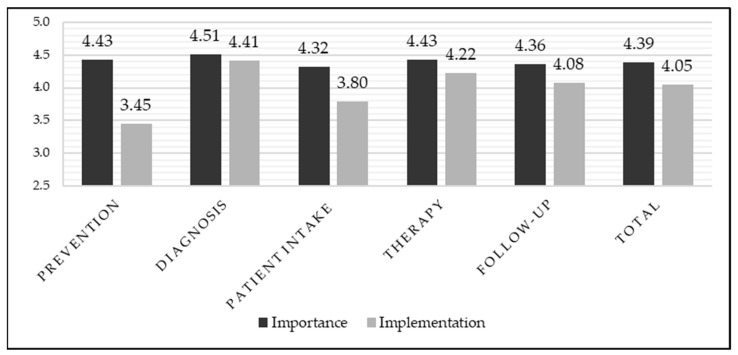
The chart illustrates the difference between the mean of perceived importance and reported implementation for the five areas of interest and for the totality of the statements. Dark grey bars are used to indicate the perceived importance of each statement (ranging from (1) “not important at all” to (5) “extremely important”), whereas light grey bars are used to show the level of reported implementation (ranging from (1) “not implemented at all” to (5) “fully implemented”).

In particular, when the mean values for the five phases are considered as a whole (Figure 2), it appears that only the prevention phase (2 statements) shows a significant gap; the patient intake phase (8 statements) is the only other phase that shows a significant discrepancy between perceived importance and reported implementation, which, however, does not exceed the “problematic” threshold of 0.75.

Analyses of the importance–implementation gap showed consistent trends across regions, with no substantial influence of regional or structural factors. In instances where a value was identified as an outlier in relation to the other regions, it was determined to be statistically non-significant due to the limited size of the subsample.

#### 3.2.4. Implementation Higher than or Equal to Importance

It is noteworthy that five statements are reported as more or as implemented as they are considered important, which may indicate a strong clinical habit and practice that sees the procedures as consolidated. These statements are reported in Table 6.

In the diagnostic phase, the initial identification and staging with ultrasound and contrast-enhanced MRI or mammography are considered of high importance and are also very highly implemented.

In the context of therapeutic interventions, the reported implementation of breast-conserving surgery (quadrantectomy) and axillary-conserving surgery (sentinel lymph node biopsy) for women diagnosed with early-stage cancer appears to be more implemented than considered important.

For the follow-up phase, the physical examination every 6–12 months from the fourth to fifth year, and the annual bilateral mammography (or contralateral if previous mastectomy) have equal scores for the mean of perceived importance and reported implementation.

## 4. Discussion

The present survey was conducted with the aim of verifying the extent of the adoption of clinical practice recommendations on TNBC management in Italian BUs. As a preliminary step, key points across the main phases of TNBC care (prevention, diagnosis, patient intake, therapy, and follow-up) were identified. BUs’ Coordinators were then invited to express the level of perceived importance and reported implementation for each item, thereby providing a measure of their perceived relevance and their application in real-world clinical settings. Overall, the findings indicate a general consensus and high reported implementation of core aspects of TNBC management among BUs, thereby providing robustness to the previously identified recommendations and supporting the development of a PJ for TNBC.

Among the aspects of care under study, only two statements presented critical reported implementation levels, registering an average score below 3, indicating a not satisfactory reported implementation, both related to issues concerning the role of GPs. The role of these professionals in the care of patients is well-established, as confirmed by the high degree of importance attributed to them by BUs’ Coordinators. They often represent the primary access point to healthcare services for patients, thereby playing a pivotal role in cancer prevention, supporting clinical decisions and providing information and indications to patients throughout their journey [26]; however, the findings suggest that the TNBC pathway of care demonstrates insufficient involvement of GPs, primarily in two aspects: collection of family history finalized to BRCA testing and coordination with multidisciplinary care teams. The presence of BRCA gPVs has been associated with a higher probability of developing TNBC and may be inherited [27]. The early detection of such gPVs enables relevant preventive measures, which are positively related to improved survival [28]. Therefore, the role of GPs in identifying at-risk patients can be particularly impactful.

The low reported implementation levels observed in the survey may be attributable to limited recognition of their role and low expertise of GPs regarding *BRCA* gPV. This finding is consistent with those of Perre et al., which highlighted the need for GP training and the contrast between the expectations of the GPs’ role according to *BRCA1/2* gPV carriers and to GPs themselves [29]. Furthermore, Wallner et al. emphasize the necessity of increasing primary care providers’ knowledge of BC treatment options [30]. It is therefore suggested that advancements in GPs’ knowledge on this topic could contribute to a reduction in this gap, thus improving the detection of at-risk populations. Moreover, it is necessary to address systemic barriers to the effective delivery of GP services, encompassing external factors that could impede successful involvement and contribution to the journey of TBNC patients. Klitzman and Chung also underscored the need for better communication between physicians and patients regarding prophylactic surgical measures [31].

Furthermore, enhanced coordination between GPs and the multidisciplinary team could contribute to more efficient patient management, thus improving communication both among the healthcare professionals and towards patients. The strengths of primary care have been acknowledged by experts, who have also underscored the significance of enhancing integrated care [32]. In Italy, initiatives and regulations aimed at implementing community-based care are currently ongoing; this organizational development may foster greater integration between healthcare services and professionals, including BUs and GPs. As a result of the discrepancy between their levels of reported implementation and perceived importance, the statements mentioned above were among the four that showed a critical gap between the two dimensions. In particular, Statement 12, focusing on ‘Coordination between the multidisciplinary team and the GP, through access to medical reports and an open channel of communication’, exhibits the most significant discrepancy in the entire survey (a gap of 1.43). This substantial gap suggests a potential need for the implementation of structured and protected communication channels (such as teleconsultations or dedicated platforms) to ensure effective continuity of care between the BUs and primary care, a key element of optimal patient journey models worldwide.

Another pivotal aspect in the patient intake phase, closely related to the coordination issues, is the presence of the case manager (Statement 7). With a perceived importance score of 4.68 and a reported implementation score of 3.96, the resulting gap of 0.72 highlights a significant operational challenge. While not exceeding the arbitrary 0.75 threshold for substantial criticality, the case manager’s role in the multidisciplinary team is globally recognized as essential for standardizing and improving the patient journey, especially for complex and heterogeneous diseases like TNBC. It is relevant to specify that, despite possible differences in professional backgrounds, the role and associated tasks of case managers are considered consistent among Breast Units, as they are defined by specific requirements and competencies.

The other two items exhibiting a critical gap pertain to waiting times and the publication of BU registers.

The statement addressing waiting times specifically focused on gynaecological services: as previously stated, timing is especially relevant with respect to implications of TNBC and its treatment. For example, *BRCA* gPVs have been linked with ovarian cancer, and preventive gynaecologic surgery may therefore be suggested. In addition, TNBC treatment can affect gynaecological health and fertility. Consequently, ensuring that patients receive timely gynaecological counselling prior to surgery and therapeutic intervention appears to be an organizational priority. Timing is widely acknowledged as a crucial factor in cancer care, and waiting times remain a common unmet need in healthcare systems across various countries.

Finally, a significant discrepancy (gap of 0.94) exists for Statement 13: “Regions should update and publish annually/biennially the lists of recognized Breast Units” (Importance 4.70, Implementation 3.76). This finding points to a significant deficit in institutional transparency. The lack of public, up-to-date registers impedes a patient’s ability to make an informed choice of high-volume, quality-assured centres. In Italy, BUs are recognized by law and must comply with codified standards of care for their accreditation. The international standard identifies the publication of these lists as essential for public accountability and for directing patients toward specialized care pathways, which is particularly vital for complex and heterogeneous diseases such as TNBC. Moreover, an observatory has been established to monitor the implementation of BU standards on a national scale. This framework leads to expectations regarding the increasing codification and systematic publication of BU registers.

Overall, notwithstanding the absence of a standard care pathway for TNBC, the results display a general level of consensus among BUs’ Coordinators regarding both the reported implementation and the perceived importance of key steps in TNBC management. Furthermore, gaps between the two dimensions are mostly within the acceptable threshold, indicating no pervasive unmet need.

However, despite the promising results, the study presents some limitations.

Firstly, the responding BUs represent only 65.4% of those invited to participate in the survey. Moreover, two regions are absent entirely: Calabria, despite our request for participation to three BUs, which did not respond, and Molise, which, at the time of writing, does not possess BUs affiliated with Senonetwork. Furthermore, while in some Regions all or most of the BUs are represented (e.g., Abruzzo, Trentino Alto Adige, Marche, Friuli-Venezia Giulia, and Valle d’Aosta’s BUs had a 100% response rate, and over 80% representation in Veneto), others exhibit a representation of half or less of the total BUs present (e.g., Liguria with 40%, Puglia with 20%, and Campania, Sardegna, and Basilicata with 50%).

Therefore, although a significant proportion of the BUs provided responses, it cannot be concluded that the data offer a comprehensive representation of the opinions and standard practices observed across all BUs in Italy. It could be worthwhile to consider the potential value in future research of filling this gap in the data, although such an endeavour may not offer significant insight, given the existing uniformity in the standards adhered to by all BUs. Moreover, the PJ delineates the standards of care delivered within the Italian public and accredited private healthcare systems and the regulatory measures supporting patients and caregivers, which are instrumental in preventing or reducing “financial toxicity” [33]. Investigating compliance with the optimal care journey may facilitate the identification of costs that may arise when such standards are not adequately implemented.

## 5. Conclusions

The co-participatory process supported the creation of a PJ, ensuring methodological robustness grounded in both clinical practice and real-world care experience. Firstly, the elaboration and selection of statements contributed to the definition of optimal care journeys for TNBC. Clinical support for the statements was then reached through a validation process, while the survey allowed for real-life evidence by comparing clinical statements with the reported reality of care in Italian BUs. Consequently, this study strengthened the patient journey (PJ) for TNBC by integrating real-world evidence, ensuring its scientific accuracy while reflecting current care practices. The resulting PJ can serve as a practical tool for patients and caregivers, providing reliable information on the current care context while also remaining comprehensible and actionable for non-professional audiences.

The PJ can be disseminated among clinicians, Breast Units, and patient associations, facilitating widespread use and patient empowerment.

Moreover, the co-participatory methodology used to develop this PJ is replicable across different conditions, demonstrating its versatility and potential for broader application in healthcare pathway development. This approach is particularly relevant to conditions with limited treatment options and lacking specific and structured care pathways, such as TNBC.

Future investigations ought to tackle the issue of incomplete data, namely by gathering information on those regions that did not participate or only partially participated. In addition, it will be necessary to verify the consistency of the statements in view of updates in good practice points and guidelines, following clinical advancements and scientific discoveries. Moreover, it will be critical to monitor effective compliance with optimal care pathways at different geographical levels in order to estimate the economic and clinical costs of non-compliance with standards. This will provide useful evidence to guide improvement strategies and the efficient allocation of resources.

Issues arising from the results, such as the potential system-level barriers limiting the role of GPs in the care journey of TNBC patients, should also be highlighted in future institutional frameworks to guide further investigation and the development of appropriate solutions.

## Figures and Tables

**Table 1:** Regional representation.

Region	Senonetwork	Sample	% Represented
Abruzzo	3	3	100.0%
Basilicata	2	1	50.0%
Calabria	3	0	0.0%
Campania	8	4	50.0%
Emilia-Romagna	12	9	75.0%
Friuli-Venezia Giulia	4	4	100.0%
Lazio	15	8	53.3%
Liguria	5	2	40.0%
Lombardy	34	26	76.5%
Marche	2	2	100.0%
Molise	0	-	-
Piedmont	15	9	60.0%
Puglia	10	2	20.0%
Sardinia	2	1	50.0%
Sicily	9	5	55.6%
Tuscany	12	9	75.0%
Trentino-Alto Adige/Südtirol	3	3	100.0%
Umbria	4	3	75.0%
Valle d’Aosta/Vallée d’Aoste	1	1	100.0%
Veneto	12	10	83.3%
Total	156	102	65.4%

**Table 2:** Statements pertaining to the team.

Number	Statement
Team	
I	Professions present in the Breast Unit (BU)
II	Frequency of BU meetings

**Table 3:** Importance of statement and degree of implementation (mean scores, standard deviations, modes, medians, and IQRs).

	Importance					Implementation				
	Prevention	Diagnosis	Patient Intake	Therapy	Follow-Up	Prevention	Diagnosis	Patient Intake	Therapy	Follow-Up
Mean	4.43	4.51	4.32	4.43	4.36	3.45	4.41	3.80	4.22	4.08
Std deviation	0.84	0.79	0.83	0.87	0.82	1.17	0.89	1.07	1.01	0.99
Mode	5	5	5	5	5	4	5	4	5	5
Median	5	5	5	5	5	4	5	4	5	4
Q1	4	4	4	4	4	2	4	3	4	4
Q3	5	5	5	5	5	4	5	5	5	5
IQR	1	1	1	1	1	2	1	2	1	1

**Table 4:** Importance, mean score < 4.

N		Importance
Diagnosis		
4	During diagnosis, PET may be recommended for patients with triple-negative carcinoma at clinical stage ≥ II.	3.89
Patient intake		
8.2	From the onset: Consultation with a cardiologist.	3.74
8.4	From the onset: The presence of a bone specialist.	3.92
8.5	From the onset: Dental assessment in cases where anti-blastic treatment is planned.	3.62
Therapy		
18	In the field of gynaecological health, for patients with triple-negative cancer, the possibility of using hormone therapy should not be overlooked.	3.63
Follow-up		
19	Follow-up management entrusted to the oncologist, with possible management by the GP one year after diagnosis (for low-risk breast cancer).	3.78

**Table 5:** Gaps.

N		Gap
Prevention		
1	Systematic medical history review by the GP for familiarity with BRCA gPVs.	1.53
Patient intake		
10	Waiting lists for gynaecological services not over 72 h.	0.75
12	Coordination between the multidisciplinary team and the GP, through access to medical reports and an open channel of communication.	1.43
13	Regions should update and publish the lists of recognized BUs annually/biannually.	0.93

**Table 6:** Statements that present a reported implementation score higher than or equal to the perceived importance score.

N		Importance	Implementation	Gap
Diagnosis				
3.2	Initial identification and staging with: Ultrasound (including axillary lymph nodes).	4.79	4.82	−0.03
3.3	Initial identification and staging with contrast-enhanced MRI or contrast-enhanced mammography.	4.55	4.59	−0.04
Therapy				
16	Breast-conserving surgery (quadrantectomy) and axillary-conserving surgery (sentinel lymph node biopsy) for women with early-stage cancer, without axillary lymph node involvement, not undergone neoadjuvant chemotherapy.	4.60	4.62	−0.02
Follow-up				
20.2	Follow-up examinations: Physical examination every 6–12 months from the fourth to fifth year	4.52	4.52	0.00
20.4	Follow-up examinations: Bilateral mammography (or contralateral if previous mastectomy) performed annually, supplemented by ultrasound and MRI if appropriate (not for bilateral mastectomy)	4.62	4.61	0.01

## Data Availability

The datasets presented in this article are not readily available due to technical limitations. Requests to access the datasets should be directed to the corresponding authors.

## Supplementary Materials

The following supporting information can be downloaded at: https://www.mdpi.com/article/10.3390/curroncol33020083/s1, Table S1: Importance of statement and degree of implementation.

## Abbreviations

The following abbreviations are used in this manuscript:

TNBC	Triple-Negative Breast Cancer
BU	Breast Unit
GPPs	Good Practice Points
BC	Breast Cancer
OS	Overall Survival
RFI	Relapse-Free Interval
GP	General Practitioner
ER	Oestrogen Receptor
PR	Progesterone Receptor
HER2	Human Epidermal Growth Factor Receptor 2
ADP	Adenosine Diphosphate
PARP	Poly ADP-Ribose Polymerase
gPV	Germline Pathogenic Variant
CDK	Cycline-Dependent kinase
PI3K/AKT/mTOR	Phosphatidylinositol 3-kinase, Protein Kinase B, Mammalian Target of Rapamycin
TKI	Tyrosine Kinase Inhibitor
PD-1	Programmed Cell Death protein 1
PD-L1	Programmed Death Ligand 1
PJ	Patient Journey
PET	Positron Emission Tomography
PDTA	Percorsi Diagnostico Terapeutici Assistenziali (Diagnostic and Therapeutic Care Pathways)
CAWI	Computer-Assisted Web Interviewing
MRI	Magnetic Resonance Imaging
