# Supplementary material for: Patient Journey for Triple-Negative Breast Cancer: Optimal Care Pathways vs. Reality of Care in Italian Breast Units
Source: Curr Oncol. 2026 Jan 31;33(2):83. doi: 10.3390/curroncol33020083 (PMC12938997; doi:10.3390/curroncol33020083)
Supplement: Supplementary file 1 [file curroncol-33-00083-s001.zip › curroncol-4082687-supplementary.pdf]

Supplementary file

Table S1. Importance of statement and degree of implementation.

Number	Statement	Importance	Implementation
	Prevention		
1	Systematic medical history review by the general practitioner for familiarity with BRCA germline pathogenic variant (gPV).	4.23	2.70
2	Women with a strong family history or gPV in the BRCA1 and/or BRCA2 genes should begin screening at 25 or 10 years before the age of diagnosis of the youngest affected family member.	4.64	4.21
	Diagnosis		
3.1	Initial identification and staging with: Mammography.	4.81	4.77
3.2	Initial identification and staging with: Ultrasound (including axillary lymph nodes).	4.79	4.82
3.3	Initial identification and staging with: Contrast-enhanced Magnetic Resonance Imaging (MRI) or contrast-enhanced mammography.	4.55	4.59
3.4	Initial identification and staging with: Ultrasound-guided needle aspiration of the most significant and accessible lymph node (if secondary involvement of axillary lymph nodes is suspected).	4.72	4.67
4	During diagnosis, Positron Emission Tomography (PET) may be recommended for patients with triple-negative carcinoma at clinical stage ≥II.	3.89	3.73
5.1	BRCA testing: It should be performed through germline testing, accompanied by counselling, aimed at identifying the risk in patients and family members and the possibility of using PARP inhibitors for treatment.	4.69	4.59
5.2	BRCA testing: May be requested electively to determine the final surgical strategy.	4.60	4.46
5.3	BRCA testing: Testing of genes other than BRCA1 and BRCA2 for a complete profile of molecular lesions.	4.33	4.26
5.4	BRCA testing: Appointment with psychologist, oncologist, breast surgeon, and plastic surgeon if the test is positive.	4.69	4.59
5.5	BRCA testing: If family members test positive, prophylactic surgery may be recommended based on family history.	4.06	3.98
6	Waiting times for tests not over two weeks for cases characterised by rapid disease progression.	4.48	4.10
	Patient intake		
7	Case manager in multidisciplinary team to coordinate the care pathway.	4.68	3.96
8.1	From the onset: Assessment by a psychologist/psycho-oncologist.	4.41	4.18
8.2	From the onset: Consultation with a cardiologist.	3.74	3.51
8.3	From the onset: Gynaecologist in relation to motherhood, sexuality and preventive menopause.	4.27	3.85
8.4	From the onset: The presence of a bone specialist.	3.92	3.55
8.5	From the onset: Dental assessment in cases where anti-blastic treatment is planned.	3.62	3.23
8.6	From the onset: Raise awareness among patients about nutrition, physical activity and healthy lifestyles with ad hoc material.	4.38	3.99

8.7	From the onset: In the case of BRCA gPV counselling on planning preventive gynaecological surgery.	4.75	4.55
8.8	From the onset: Palliative care specialist to manage treatment toxicity, severe pain and symptoms during transitions.	4.18	3.74
9	Information on social egg freezing.	4.75	4.31
10	Waiting lists for gynaecological services not over 72 hours.	4.39	3.64
11	Identify a codified and shared set of criteria concerning overweight, obesity, weight gain/loss and malnutrition.	4.02	3.58
12	Coordination between the multidisciplinary team and the General Practitioner (GP) through access to medical reports and an open channel of communication.	4.40	2.97
13	Regions should update and publish annually/biennially the lists of recognised Breast Units.	4.70	3.76
14	Involvement of associations to work in synergy with breast units and local services.	4.61	4.14
	Therapy		
15	Inclusion of immediate reconstruction at the same time as the demolitive surgery in the evaluation of the surgical plan.	4.82	4.68
16	Breast-conserving surgery (quadrantectomy) and axillary-conserving surgery (sentinel lymph node biopsy) for women with early-stage cancer, without axillary lymph node involvement, not undergone neoadjuvant chemotherapy.	4.60	4.62
17	Where/when known, it is important to refer patients to clinical trials in order to offer them additional treatment options.	4.66	4.25
18	In the field of gynaecological health, for patients with triple-negative cancer, the possibility of using hormone therapy should not be overlooked.	3.63	3.34
	Follow-up		
19	Follow-up management entrusted to the oncologist, with possible management by the GP one year after diagnosis (for low-risk breast cancer).	3.78	3.41
20.1	Follow-up examinations: Physical examination every 3–6 months during the first 3 years.	4.28	4.25
20.2	Follow-up examinations: Physical examination every 6–12 months from the fourth to fifth year.	4.52	4.52
20.3	Follow-up examinations: Annual physical examination after the fifth year.	4.31	4.23
20.4	Follow-up examinations: Bilateral mammography (or contralateral if previous mastectomy) performed annually, supplemented by ultrasound and MRI if appropriate (not for bilateral mastectomy).	4.62	4.61
21	Intensive follow-up not recommended in the absence of individual clinical suspicions or personalised programmes.	4.33	4.25
22.1	Follow-up services: Sexual health counselling and fertility preservation, if necessary.	4.44	3.86
22.2	Follow-up services: Genetic counselling.	4.60	4.32
22.3	Follow-up services: Bone health counselling.	4.39	3.96
22.4	Follow-up services: Counselling for nutritional education.	4.41	3.82
22.5	Follow-up services: Counselling for cardiac monitoring.	4.13	3.61
22.6	Follow-up services: Education on recognising symptoms of oncological relevance in terms of reoccurrence and late toxicity.	4.50	4.09

Table S1. The table shows the mean of importance, i.e, the degree to which the respondents perceive a Statement as important on a scale from (1) “of no importance” to (5) “extremely important”, and implementation, the reported implementation of the Statement from (1) “not implemented at all” to (5) “extremely implemented”, for each Statement.
